# Data Mining in Bioinformatics (BIOKDD)

**DOI:** 10.1186/1748-7188-2-4

**Published:** 2007-04-11

**Authors:** Mohammed J Zaki, George Karypis, Jiong Yang

**Affiliations:** 1 Computer Science Department, Rensselaer Polytechnic Institute, Troy, NY 12180, USA; 2 Department of Computer Science, University of Minnesota, Minneapolis, MN 55455, USA; 3 Electrical Engineering and Computer Science Department, Case Western Reserve University, Cleveland, OH 44106, USA

Data Mining is the process of automatic discovery of novel and understandable models and patterns from large amounts of data. Bioinformatics is the science of storing, analyzing, and utilizing information from biological data such as sequences, molecules, gene expressions, and pathways. Development of novel data mining methods will play a fundamental role in understanding these rapidly expanding sources of biological data.

Data mining approaches seem ideally suited for bioinformatics, which is data-rich, but lacks a comprehensive theory of life's organization at the molecular level. The extensive databases of biological information create both challenges and opportunities for developing novel data mining methods. The 6th Workshop on Data Mining in Bioinformatics (BIOKDD) was held on August 20th, 2006, Philadelphia, PA, USA, in conjunction with the 12th ACM SIGKDD International Conference on Knowledge Discovery and Data Mining. The goal of the workshop was to encourage KDD researchers to take on the numerous challenges that Bioinformatics offers. The BIOKDD workshops have been held annually in conjunction with the ACM SIGKDD Conferences, since 2001. Additional information about BIOKDD can be obtained online [1].

Five revised and expanded papers were selected from the BIOKDD workshop, out of a total of 18 submissions, to appear in Algorithms for Molecular Biology (AMB). These papers underwent another round of external reviewing prior to being accepted for AMB. An overview of each paper is given below. In the paper titled *Automatic Layout and Visualization of Biclusters*, Gregory A. Grothaus, Adeel Mufti and T. M. Murali [2], present a novel method to display biclusters mined from gene expression data. The approach allows querying and visual exploration of the clusters/sub-matrices. The software is also available as open-source.

In *ExMotif*: *Efficient Structured Motif Extraction*, Yongqiang Zhang and Mohammed J. Zaki [3], describe a new algorithm called EXMOTIF to extract frequent motifs from DNA sequences. The method can mine structured motifs and profiles which have variable gaps between different elements. The demonstrate the efficiency of the method compared to state-of-the-art methods, and also demonstrate an application in mining composite transcription factor binding sites.

In the paper *Refining Motifs by Improving Information Content Scores using Neighborhood Profile Search*, Chandan K. Reddy, Yao-Chung Weng and Hsiao-Dong Chiang [4], show how one can refine the profile motifs discovered via Expectation Maximization and Gibbs Sampling based methods. They search the neighborhood regions of the initial alignments to obtain locally optimal solutions, which improve the information content of the discovered profiles.

In their paper, *A Novel Functional Module Detection Algorithm for Protein-Protein Interaction Networks*, Woochang Hwang, Young-Rae Cho, Aidong Zhang and Murali Ramanathan [5], describe the unexpected properties of the protein-protein interaction (PPI) networks and their use in a clustering method to detect biologically relevant functional modules. They propose a new method called STM (signal transduction model) to detect the PPI modules, and compare it with previous approaches to demonstrate its effectiveness in discovering large and arbitrary shaped clusters.

In *A Spatio-temporal Mining Approach towards Summarizing and Analyzing Protein Folding Trajectories*, Hui Yang, Srinivasan Parthasarathy and Duygu Ucar [6], describe a method to mine protein folding molecular dynamics simulations datasets. They describe a spatio-temporal association discovery approach to mine protein folding trajectories, to identify critical events and common pathways.
